# Tissue Constructs with Human Adipose-Derived Mesenchymal Stem Cells to Treat Bone Defects in Rats

**DOI:** 10.3390/ma12142268

**Published:** 2019-07-15

**Authors:** Guilherme Caetano, Weiguang Wang, Adriana Murashima, José Roberto Passarini, Leonardo Bagne, Marcel Leite, Miguel Hyppolito, Salem Al-Deyab, Mohamed El-Newehy, Paulo Bártolo, Marco Andrey Cipriani Frade

**Affiliations:** 1 Department of Internal Medicine, Ribeirão Preto Medical School, University of São Paulo (USP), Ribeirão Preto 14040-900, SP, Brazil; 2 Graduate Program in Biomedical Sciences, University Centre of Hermínio Ometto Foundation, Araras 13607339, SP, Brazil; 3 School of Mechanical, Aerospace and Civil Engineering, University of Manchester, Manchester M13 9PL, UK; 4 Department of Ophthalmology, Otolaryngology and Head and Neck Surgery, Ribeirão Preto Medical School, University of São Paulo (USP), Ribeirão Preto 14040-900, SP, Brazil; 5 Department of Chemistry, College of Science, King Saud University, Riyadh 11451, Saudi Arabia; 6 Department of Chemistry, Faculty of Science, Tanta University, Tanta 31527, Egypt

**Keywords:** adipose-derived stem cell, bone repair, biofabrication, tissue engineering

## Abstract

The use of porous scaffolds created by additive manufacturing is considered a viable approach for the regeneration of critical-size bone defects. This paper investigates the xenotransplantation of polycaprolactone (PCL) tissue constructs seeded with differentiated and undifferentiated human adipose-derived mesenchymal stem cells (hADSCs) to treat calvarial critical-sized defect in Wistar rats. PCL scaffolds without cells were also considered. In vitro and in vivo biological evaluations were performed to assess the feasibility of these different approaches. In the case of cell seeded scaffolds, it was possible to observe the presence of hADSCs in the rat tissue contributing directly (osteoblasts) and indirectly (stimulation by paracrine factors) to tissue formation, organization and mineralization. The presence of bone morphogenetic protein-2 (BMP-2) in the rat tissue treated with cell-seeded PCL scaffolds suggests that the paracrine factors of undifferentiated hADSC cells could stimulate BMP-2 production by surrounding cells, leading to osteogenesis. Moreover, BMP-2 acts synergistically with growth factors to induce angiogenesis, leading to higher numbers of blood vessels in the groups containing undifferentiated and differentiated hADSCs.

## 1. Introduction

Tissue engineering comprising scaffold-based and cell laden approaches represents a viable alternative to conventional grafting approaches [1,2,3,4,5,6,7]. These different approaches have been explored to treat a wide range of tissues including bone, cartilage, muscle, nerve and skin [8,9,10,11,12,13,14,15,16,17].

In the case of bone, the scaffold-based approach is the most commonly used. It is based on the use of 3D interconnected biocompatible and biodegradable porous structures that provide the correct biomechanical environment for cell attachment, proliferation and differentiation [6,16,17]. Different materials (natural and synthetic polymers, ceramics and polymer/ceramic composites) and fabrication techniques (additive manufacturing and non-additive manufacturing processes) were explored [5,15,16,18]. However, there is still a significant debate regarding the best material composition and scaffold topology for specific tissue engineering applications. The implantation of scaffolds without cells or seeded with undifferentiated or differentiated cells is also being investigated in order to identify the most suitable approach.

Poly(ɛ-caprolactone) (PCL) is a semi-crystalline biodegradable polymer which is widely used for bone tissue engineering applications due to its biocompatibility, non-cytotoxicity, favorable mechanical and degradation properties [4,19]. However, PCL is a hydrophobic material with low bioactivity, which compromises cell attachment, osteoconduction and osteoinduction. In order to overcome some limitations, our research group previously reported the use of interconnected PCL scaffolds, which were NaOH-treated, for human adipose-derived mesenchymal stem cell (hADSC) proliferation and differentiation into osteoblasts-like cells [8,20,21,22]. No toxicity effects were observed, and the treatment did not induce any physical changes on the scaffold characteristics. Preliminary in vivo results were published by our group focusing on the use of PCL scaffolds seeded with osteoblasts and mesenchymal stem cells to treat critical-size bone defects created in the calvaria bone of male Wistar rats, showing a positive impact of the combined used of PCL scaffolds with mesenchymal stem cells in terms of new bone formation [21].

In this paper, we expand upon this initial study, providing a more in-depth analysis on the in vivo role of human adipose-derived mesenchymal stem cells (pre-in-vitro differentiated and undifferentiated) seeded on PCL scaffolds. Masson’s trichrome staining is used to assess the tissue formation [23]. Immunohistochemistry analysis is conducted in order to investigate the role of hADSC on the rat tissue formation and to quantify different growth factors to evaluate both bone repair stimulation and angiogenesis.

## 2. Materials and Methods

### 2.1. Scaffold Design and Fabrication

PCL (CAPA 6500, Mw ≈ 50,000 Da, Perstorp, Cheshire, UK) scaffolds were produced using the screw-assisted additive manufacturing system 3D Discovery (RegenHU, Villaz-Saint-Pierre Switerzland). A lay-down pattern of 0°/90° was adopted to obtain pores with a regular square geometry and a constant filament distance of 680 μm. Large scaffolds blocks (30 mm × 30 mm × 6 mm) were produced using a melting temperature of 90 °C, slice thickness of 220 μm, screw rotation velocity of 22 rpm, and deposition velocity of 20 mm/s. After fabrication, scaffold samples were cut with fine double-edged razor blades into small blocks (~11 mm × 11 mm × 6 mm) to fit in a 24-well culture plate for in vitro studies. Scaffolds were surface-treated by soaking them in 5M NaOH for 3 h, rinsed with phosphate buffered saline (PBS, pH 7.4, (Sigma-Aldrich, Dorset, UK)), sterilized in 70% ethanol for 24 h, rinsed with PBS and air-dried.

### 2.2. In Vitro Cell Proliferation, Differentiation and Bioimaging

Human adipose-derived stem cells (hADSCs) (StemPro^®^, Invitrogen, Carlsbad, CA, USA) were used for in vitro studies. The cell viability/proliferation on PCL scaffolds was assessed considering 14 days of cell culture. In order to determine the osteogenic differentiation of hADSCs cultured on scaffolds, cell differentiation tests were also performed for 21 days of cell culture with StemPro^®^ Osteocyte/Chondrocyte Differentiation Basal Media (Invitrogen, Carlsbad, CA, USA). Three scaffold samples were used in each test, and all experiments had at least three scientific repeats. In both cases, on the last day of cell culture, scaffolds were assessed using scanning electron microscopy (SEM) and confocal microscopy. For SEM analysis, scaffolds were fixed with a 3% glutaraldehyde solution (Sigma-Aldrich, UK) for 30 min at room temperature, rinsed twice with PBS, dehydrated with a graded ethanol series (50%, 70%, 80%, 90%, and 100% (twice)), in 50:50 ethanol/hexamethyldisilazane (HMDS, Sigma-Aldrich, UK) and then in 100% HMDS (with 10 min exposure at each step), and finally air dried for HMDS removal. Thin cross-section layers of each sample (around 1 mm) were cut and platinum-coated for imaging using a Gatan Model 682 Precision Etching Coating System, to an approximate thickness of 7 nm. SEM images were obtained using a Hitachi S300N microscope (Hitachi, Tokyo, Japan) [8,20]. In the case of confocal microscopy analysis, samples were removed from the cell culture plate, rinsed twice in PBS, fixed using 4% paraformaldehyde for 40 min, and then washed twice with PBS prior to immersion for 30 min in an immunocytochemistry blocking buffer comprised of 2% goat serum and 1% bovine serum albumin in PBS. Samples were again rinsed twice in PBS. Cell nuclei were stained blue by soaking them in a PBS solution containing 4,6-Diamidine-2′-phenylindole dihydrochloride (DAPI) (Invitrogen™, ThermoFisher Scientific, Waltham, MA, USA) at the manufacturer’s recommended concentration, then cell actin-stained using Alexa Fluor™ 488 Phalloidin (Invitrogen™, ThermoFisher Scientific, Calsbad, CA, USA) diluted to the manufacturer recommended level. Samples were left in the staining solution for 10 min prior to removal and rinsed twice thoroughly with PBS. Confocal images were obtained on a Leica TCS SP5 (Leica Microsystems, Mannheim, Germany) confocal microscope.

### 2.3. Cell Source and Culture for In Vivo Studies

Human adipose-derived mesenchymal stem (hADSCs) cells for in vivo studies were isolated from patients from Ribeirão Preto Medical School (Brazil), following a procedure approved by the the hospital’s ethical committee (number 2722/2014). The extracellular matrix (ECM) of lipoaspirate adipose tissue was digested in 0.075% collagenase (Sigma-Aldrich, UK) for 30 min at 37 °C. The digested adipose tissue was centrifuged and then plated in a tissue culture plate. Cells were cultured and expanded at 37 °C under 5% CO_2_ using a minimum essential medium (α-MEM) plus 10% fetal bovine serum, 1% antibiotic-antimycotic, 1% l-Glutamine 200 mM, all Invitrogen). The medium was changed twice per week, and cells were passaged on reaching 80–90% confluence by the use of 0.25% trypsin-EDTA solution (Invitrogen, USA). Passages 3 to 5 were used for this study.

### 2.4. hADSC Characterization and Multilineage Differentiation

A BD-FACSCalibur cytometer (BD, Becton-Dickson) was used with specific fluorescein-conjugated monoclonal antibodies including CD29, CD44, CD73, CD90, CD105 (positive markers) and CD14, CD31, CD34, CD45, CD71 CD144, CD11b, and Anti-HLA (negative markers) (B&D Bioscience, San Jose, CA, USA). After culturing cells on 24-well plates, adherent cells were harvested (*n* = 4) and followed to incubation with antibodies for 20 min and washed twice with PBS. Data was analyzed with Cellquest software and presented as means of CD marker percentage values.

hADSCs were incubated in α-MEM basic medium supplemented with 10 µg/mL of insulin (Sigma-Aldrich, UK), 100 µM of indomethacin (Sigma-Aldrich, UK), and 1 µM of dexamethasone (Sigma-Aldrich, UK) for adipogenic differentiation (*n* = 4). Moreover, hADSCs were incubated in α-MEM basic medium supplemented with 200 µM of ascorbic acid (Sigma-Aldrich, UK), 10 mM of β-glycerolphosphate (Sigma-Aldrich, UK) and 0.5 µM of dexamethasone (Sigma-Aldrich, UK) for osteogenic differentiation (*n* = 4). The medium of both cultures was replaced every 3 days over 21 days. Sudan IV and Alizarin Red-S (ARS) were used to evaluate the presence of cell differentiation.

### 2.5. Cell Seeding for In Vivo Studies

Scaffolds were seeded with 5 × 10^4^ hADSCs in 50 µL of α-MEM basic media. The cells used were at passage 5, and cells from only one donor were used. Cell-seeded scaffolds were cultivated at standard conditions over 2 h, allowing cells to diffuse and adhere into the scaffold. Two different cell culture media were considered: basic culture media and osteogenic media (basic culture media supplemented with 200 μM of ascorbic acid, 10 mM of β-glycerophosphate and 0.5 μM of dexamethasone), used in separated 24-well plates. The media was changed every 3 days for 21 days, creating tissue constructs with hADSCs prior to implantation in the bone defects. Additionally, scaffolds were kept in a separated 24-well plate containing basic media with no cells.

### 2.6. Bone Defect Creation

Critical bone defects were created in the calvaria bone of male Wistar rats (with weights of 250–300 g), according to the ethical guidelines of the Brazilian College of Animal Experimentation, approved by the Ethical Committee on Animal Experimentation from Ribeirão Preto Medical School, University of São Paulo (number 024/2015-1). Sixteen animals were anesthetized with xylazine hydrochloride (10 mg/kg) and ketamine hydrochloride (30 mg/kg), and a circular bone defect (5 mm in diameter) was created. Briefly, after shaving the hair over the calvaria and asepsis with 70% ethanol, a circular bone defect was created on the right side of the calvarial bone using a trephine drill at 1200 rpm with constant PBS irrigation.

Four groups were considered (*n* = 4 animals/group): natural bone repair without a scaffold (NBR); PCL scaffold without cells (SCA); PCL scaffolds with hADSCs cultivated in basic media—undifferentiated cells (SUC); PCL scaffolds with hADSCs cultivated in osteogenic media—pre-differentiated cells (SDC). The animals were euthanized after 60 days using excessive anesthetic intraperitoneal administration, and bone defect samples (biopsies) were collected for further analysis.

### 2.7. Histology

Bone biopsies were fixed in 3.7% buffered formaldehyde solution (pH 7.4) for 48 h and demineralized with 10% ethylenediaminetetraacetic acid for 30 days. Biopsies were then dehydrated, embedded in paraffin, and then 5.0 μm-thick cross-sections were cut and stained with Masson′s Trichrome (TM). Masson’s Trichrome staining is used to assess the tissue formation considering the connective formation, mostly by the presence of collagen, and also to reveal the degree of bone mineralization [8,23,24,25,26]. The histological sections were evaluated by light field microscopy, and the images were acquired using a LEICA DM 4000BVR microscope equipped with a LEICA DFCVR 280 camera (Leica Microsystems, Wetzlar, Germany) at 50× and 200× magnifications.

### 2.8. Immunohistochemistry

The following antibodies were considered: human anti-mitochondrial (MIT) to evaluate the presence of hADSCs in the rat bone tissue; anti-BMP-2 (bone morphogenetic protein-2) to evaluate bone repair stimulation; and anti-CD31 for the angiogenesis effect (ABCAM, Cambridge, MA, USA), following the manufacturer’s recommendation. Samples were then incubated with secondary antibodies for 30 min. Liquid 3,3′-Diaminobenzidine (Springer-DAB-125) was used as substrate-chromogen for 5 min at room temperature to reveal markings and counterstained with Harris Hematoxylin for 1 min. Biopsies were analyzed using a LEICA DM 4000BVR microscope equipped with a LEICA DFCVR 280 camera and images were captured at 400× magnification. BMP-2 and CD31 images (six images per animal) were used for positive marker quantification using ImageJ software (version 1.46) (with the color deconvolution plugin) [27].

## 3. Results

### 3.1. 3D Printed Scaffolds

**Figure 1:**
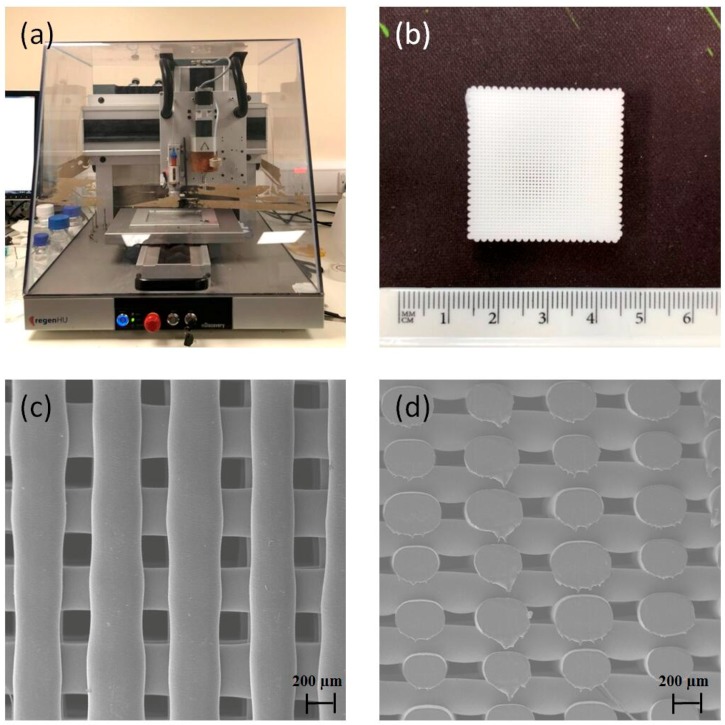
(**a**) Screw-assisted additive manufacturing system 3D Discovery; (**b**) polycaprolactone (PCL) printed scaffold. SEM images of PCL scaffolds: (**c**) top view and (**d**) cross-section view.

Extrusion-based additive manufacturing (Figure 1a) was used to print 3D PCL scaffolds (Figure 1b) with uniformly distributed regular pores (Figure 1c,d). Printed scaffolds present around 330 μm of filament diameter and 350 μm of pore size, which are similar to the initial designed values. Scaffolds present 45% porosity.

### 3.2. In Vitro Cell Proliferation, Differentation

**Figure 2:**
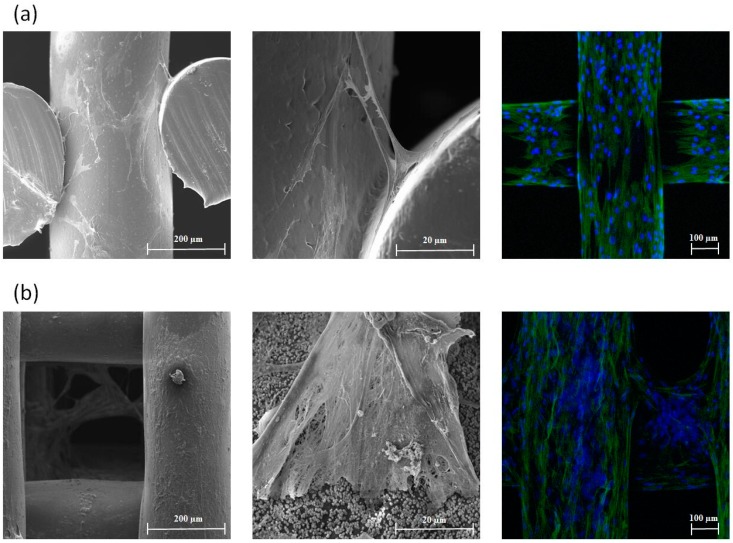
(**a**) SEM images (left and middle) and confocal microscopy image (right) for human adipose-derived mesenchymal stem cell (hADSC)-seeded scaffolds 14 days after cell proliferation; (**b**) SEM images (left and middle) and confocal microscopy image (right) for hADSC-seeded scaffolds 21 days after cell differentiation.

Preliminary bioimaging analysis (Figure 2) shows that the printed scaffolds are able to support the attachment, proliferation and differentiation of hADSCs. Confocal images also showed a significant spread of cells. Proliferated hADSCs present a spindle shape with a long extension morphology, while a fibrillary extracellular matrix network formed by calcium deposition can be observed for osteogenic differentiated hADSCs.

### 3.3. hADSCs Characterization for In Vivo Study

**Figure 3:**
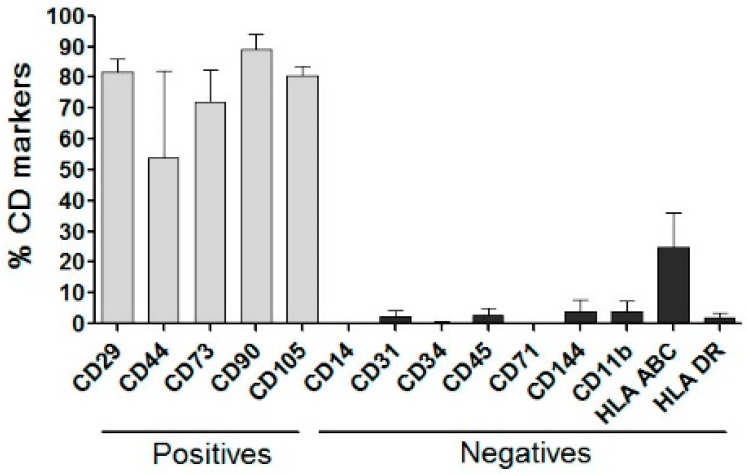
Flow cytometry of hADSCs incubated with positive and negative surface markers.

hADSCs from four different donors were analyzed by flow cytometry using cell surface markers. The results are presented in Figure 3 as percentages of staining. Results show that hADSCs presented greater expression for all positive surface markers (>80%) and low expression for the negative markers (<8%), except for anti-HLA ABC (Class I MHC marker), which is responsible for transplant rejection related to donors’ cells, which expressed at around 20%.

**Figure 4:**
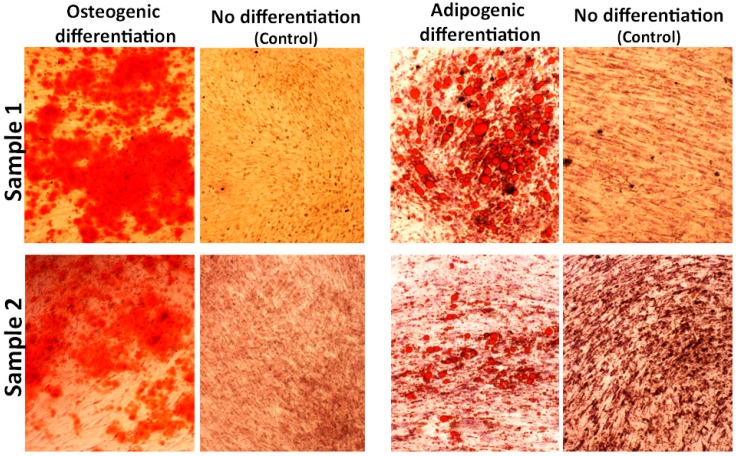
Photomicrography of hADSCs after 21 days for osteogenic differentiation, adipogenic differentiation and no differentiation (control).

To study the multilineage potential of hADSCs, cells were differentiated toward the adipogenic and osteogenic lineages using lineage-specific induction conditions for 21 days. Osteogenic differentiation was detected by Alizarin Red-S staining while the adipogenic differentiation was detected by Sudan IV, as shown in Figure 4. The osteogenesis is based on ECM mineralization related to calcium content, and the adipogenesis is based on the lipid droplet accumulation into the cells. Control cultures were added to the experiment using only basic culture medium, where there was no cell differentiation.

### 3.4. Histological Assessment

**Figure 5:**
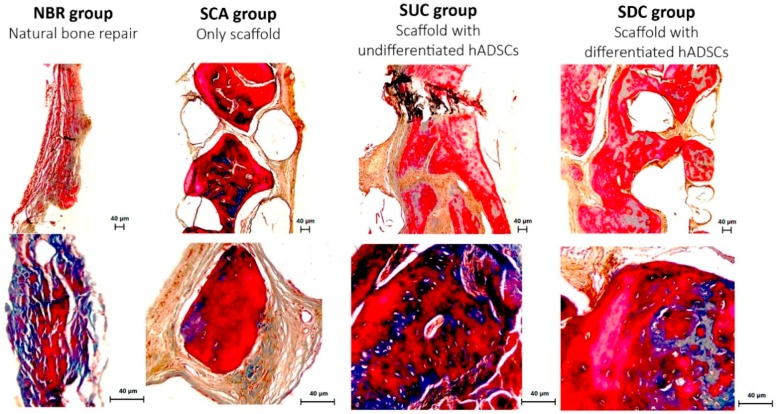
Photomicrography of the bone defect after 60 days, Masson’s Trichrome staining, at 50× and 200× magnification. Collagen connective and osteoid tissues are observed in blue and red color respectively.

Masson’s Trichrome staining is able to reveal the degree of mineralization of new tissue. The reddish color indicates mineralized tissue, while the bluish color indicates the presence of collagen, cartilage and the mineralization process, according to its organization and intensity. Figure 5 shows histological images at 50× and 200× magnifications after 8 postoperative weeks for all four groups.

Thin layers of connective tissue between the bone edges of the defect were observed in the NBR group, mainly presenting bluish collagenous connective tissue. In the SCA group, it is possible to observe the presence of “islands” of reddish mineralized tissue (osteoid) in the interconnected porous scaffold. For the groups treated with hADSC-seeded PCL scaffolds, larger osteoid tissue with a higher number of osteocytes, embedded in an organized connective tissue in the interconnected porous scaffold, is observed throughout the bone defect.

### 3.5. Immunohistochemical Assessment

**Figure 6:**
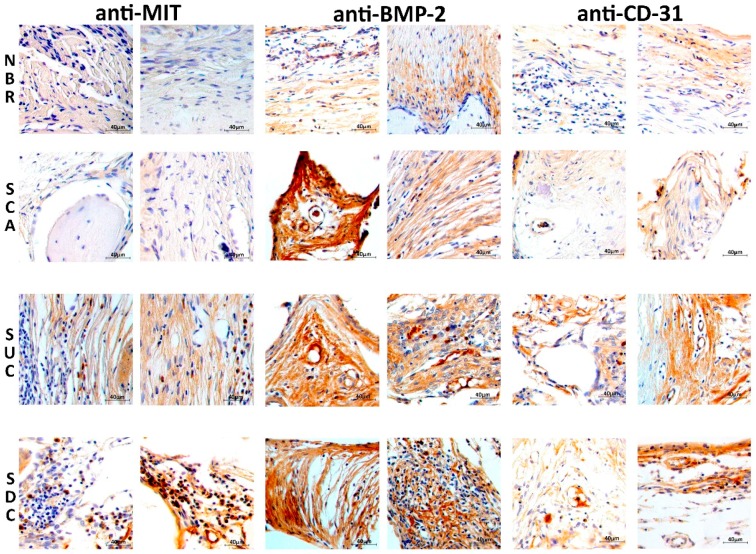
Photomicrography of the rat bone defect after 60 days at 400× magnification for the four considered groups. The histological samples were immunolabeled with human anti-mitochondrial (MIT), anti-BMP-2 (bone morphogenetic protein-2), and anti-CD-31 (blood vessel).

Figure 6 presents histological sections of the rat bone defect immunolabeled with (anti-MIT), anti-BMP-2 and anti-CD31. Images were acquired from the center area of the bone defect, considering the tissue layer in the NBR group and from the porous scaffold. The presence of human adipose-derived mesenchymal stem cells is strongly stained in a brown-ocher color (anti-MIT) in both SUC and SDC groups. It is also possible to observe the presence of the anti-BMP-2 brown-ocher staining in all groups. In the NBR group, the brown-ocher color is not as evident as the in other groups as it exists only on a thin connective tissue. Blood vessel formation (angiogenesis) represented by the anti-CD-31 label is also more evident in the groups that received scaffolds as tissue substitute.

**Figure 7:**
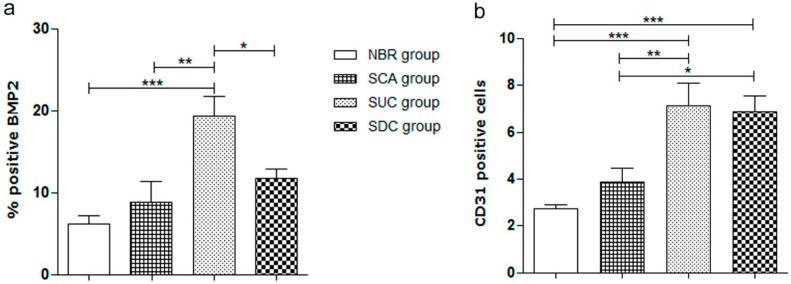
Quantification of positive anti-BMP-2 (bone morphogenetic protein-2); and anti-CD31 (blood vessel) in the bone defect area after 60 days for all considered groups. NBR—animals submitted to Natural Bone Repair without a scaffold; SCA—animals submitted to PCL SCAffold without cells; SUC—animals submitted to PCL Scaffolds with Undifferentiated Cells (hADSCs cultivated only in basic media); and SDC—animals submitted to PCL scaffolds with Differentiated Cells (hADSCs cultivated in osteogenic media). The values were compared using ANOVA and Tukey’s post-test (* *p* < 0.05; ** *p* < 0.01; *** *p* < 0.001). Results expressed as mean ± SEM.

After the quantitative evaluation of the positive expression of BMP-2 and blood vessels (CD31), it possible to observe that the SUC group presents a higher percentage of BMP-2 positive expression (*p* < 0.05) after 60 days of bone repair in comparison to the other three groups. The results also show that both SUC and SDC groups present a high number of blood vessels compared to the NBR and SCA groups (*p* < 0.05), as shown in Figure 7.

## 4. Discussion and Conclusions

Screw-assisted additive manufacturing allowed the production of scaffolds with regular pore distribution and a well-defined fiber diameter and pore size. Three-dimensional-printed PCL scaffolds were able to support hADSCS attachment, proliferation and differentiation, indicating that the processing conditions considered in this study do not induce any chemical transformation on the material, which shows no cytotoxicity. In order to create a tissue construct, hADSCs were isolated in vitro from human fat tissue and underwent a characterization process by an immunomarker panel and osteogenic/adipogenic differentiation. The characterization results show the success of the primary cell culture isolation and the maintenance of hADSCs in vitro, once the immunophenotypic and multipotential profile were reported, as already described in the literature and recommended by the International Society of Cell Therapy [28,29,30].

Although the autografts are still considered the gold standard for bone repair, the allografts are suggested to have a practical impact in future clinical applications, especially for the bone treatment of elderly patients, as cell proliferation and differentiation are age-dependent [31,32]. The major problem related to allografts is the possibility of rejection, which compromises its use in clinical trials and, for this reason, the need for further studies to increase the safety and efficacy of the long-term use of hADSCs has been emphasized [33,34]. The hADSCs used in this study had a low immunogenic profile and did not express class II histocompatibility complex molecules. Considering future clinical applications, this study aimed to provide a more in-depth analysis of the in vivo role of differentiated and undifferentiated hADSCs seeded on PCL scaffolds in an animal model of calvaria bone defect.

In vivo results show that the use of hADSC-seeded PCL scaffolds has a positive impact on tissue formation, organization and mineralization after grafting (xenotransplantation). These results corroborate to preliminary findings by Caetano et al. [21] investigating in more detail the effect of different scaffold-based approaches with and without cells to treat large bone defects. Results show the use of human ADSCs contribute directly (tissue formation) and indirectly (stimulation by paracrine factors) for rat tissue formation, organization and mineralization, as also described elsewhere [33,35,36].

Bone marrow-derived human mononuclear cells (hMSCs) remarkably promoted the calvarial bone regeneration in the immunocompetent rats, and hMSCs were able to evade the immune surveillance initially (around 10 days) [35]. The authors suggested the use of hMSCs appears to be restricted to autologous or allogeneic transplantation because the longer permanence in the defected site of tissue. Our results corroborate this partially; cell-seeded groups also stimulated bone formation, but in our study, human cells were found in the rat tissue after 60 days.

The use of human mesenchymal stem cell enables the bone reconstruction of a calvarial defect model and may have superior potential for bone reconstruction compared to osteoblast-like cells, according to morphological observations and histochemical analysis [36]. Corroborating our findings, the authors observed that the animals that received scaffolds seeded with human mesenchymal stem cells had greater bone regeneration compared to scaffolds with pre-differentiated cells. After 10 weeks, human cells were also found in the animal tissue. Although more studies are necessary in the tissue engineering field using mesenchymal stem cells, the literature encourages the use of these cells to stimulate host cells surrounding bone defects in order to enhance bone [32] and osteochondral tissues [33].

The presence of BMP-2 in the rat tissue treated with cell-seeded PCL scaffolds suggests that the paracrine factors from hADSCs might be able to stimulate BMP-2 production, leading to osteogenesis. During the bone healing process, BMPs are produced by stem cells and osteoblasts from the surrounding tissue, which seems to stimulate their proliferation and differentiation. A greater presence of BMP-2 in the animal tissue using scaffolds was also reported [35]. In our study, we suggest that the presence of transplanted hADSCs in the rat tissue, detected by the MIT marker, was able to stimulate BMP-2 release by host cells, contributing to accelerated tissue mineralization and organization.

Moreover, it is also known that BMP-2 acts synergistically with growth factors to induce angiogenesis, leading to new blood vessel formation, which strongly benefits the regenerative process [37,38,39]. As observed, the use of mesenchymal stem cells had a positive impact not only on the synthesis of BMP-2 but also on the blood vessel formation (providing oxygen and nutrients to the inner regions of the scaffold) which is directly related to the osteoblast formation and osteoclast recruitment, promoting bone regeneration and consolidation. Kaigler et al. [40] reported that the endothelial cells produce BMP-2, which corroborates our findings considering that more blood vessels were quantified in the bone defect area after 60 days. Sartori et al. [41] implanted scaffolds seeded with human mesenchymal stem cells in a subcutaneous implant model in immunocompetent animals. The authors reported stimuli of human cells for angiogenesis. In our study, the properties of the hADSCs, together with the higher presence of BMP-2, seem to have favored the formation of new blood vessels, as demonstrated by immunohistochemistry with anti-CD31 antibody in the tissue in formation, and also as reported by Huang et al. [7].

Despite the relative low porosity of the printed scaffolds (at around 40%), the results show that they provide enough internal space for angiogenesis and consequently osteogenesis. Results also suggest that the designed scaffolds with mesenchymal stem cells represent a viable approach to treat critical-size bone defects due to the immunosuppressive and regenerative properties and well-described paracrine effects. However, more studies should be conducted in order to evaluate the use of hADSCs for long periods of time and possible immunological reactions.
